# Genetic Predisposition to Excess Body Weight and Survival in Women Diagnosed With Breast Cancer

**DOI:** 10.1001/jamanetworkopen.2025.53687

**Published:** 2026-01-13

**Authors:** Clara Bodelon, Mariah Landry, Adriana Lori, James M. Hodge, Parichoy Pal Choudhury, Erika Rees-Punia, Ying Wang, Lauren E. McCullough, Alpa V. Patel, Lauren R. Teras

**Affiliations:** 1 Department of Population Science, American Cancer Society, Atlanta, Georgia; 2 Department of Surveillance, Prevention & Health Service Research, American Cancer Society, Atlanta, Georgia; 3 Emory University Rollins School of Public Health, Atlanta, Georgia

## Abstract

**Importance:**

Excess body weight, which is associated with poor survival after breast cancer (BC) diagnosis, is a heritable trait.

**Objective:**

To investigate whether genetic predisposition to excess body weight is associated with the risk of mortality among BC survivors.

**Design, Setting, and Participants:**

This cohort study is part of the Cancer Prevention Study–II Nutrition Cohort, a large study in which participants responded to a survey in 1992 and to biennial follow-up surveys starting in 1997. The cohort includes adults residing in 21 US states. Women diagnosed with a first primary nonmetastatic BC between 1992 and 2017 with genetic data were included in this study. Analyses were restricted to postmenopausal women at the time of cancer diagnosis who had genetically determined European ancestry. Data analysis was conducted from July 2023 to July 2025.

**Exposure:**

A polygenic score for body mass index (BMI-PGS), computed using summary statistics from 941 single nucleotide variants reported in a meta-analysis of genome-wide association studies that included approximately 700 000 individuals.

**Main Outcomes and Measures:**

Deaths through 2020 were identified via linkage with the National Death Index. Cox proportional hazards regression models were used to calculate hazard ratios (HRs) for the association between BMI-PGS and all-cause mortality.

**Results:**

This analysis included 4177 women diagnosed with BC. The median (IQR) age at diagnosis was 71.5 (66.3-76.7) years. BC survivors with a BMI-PGS in the top tertile were more likely to have a BMI of 30 or greater (345 [24.8%]) compared with survivors in the lowest tertile (172 [12.4%]). During a median (IQR) follow-up time of 14.5 (9.7-19.7) years, 2114 BC survivors (50.6%) died. Compared with BC survivors in the lowest tertile of the BMI-PGS, those in the highest tertile had a 15% increased risk of all-cause mortality (HR, 1.15, 95% CI, 1.04-1.28). BC survivors with BMI-PGS in the highest tertile needed to walk approximately 1.7 hours per week more to be at a similar risk level as BC survivors in the lowest tertile of the BMI-PGS, which corresponds to approximately an extra 15 minutes of walking each day of the week.

**Conclusions and Relevance:**

In this cohort of nonmetastatic BC survivors, women who were genetically predisposed to having a higher BMI were at increased risk of all-cause mortality. Targeted lifestyle recommendations to mitigate their genetic predisposition should be considered to lower this risk.

## Key Points

**Question:**

Is genetic predisposition to excess body weight associated with increased risk of mortality in breast cancer survivors?

**Findings:**

In this cohort study of 4177 breast cancer survivors, those with a polygenic score for body mass index in the top tertile had a 15% higher mortality risk compared with those in the bottom tertile of the score. A greater number of hours of walking per week was needed to mitigate the inherited susceptibility.

**Meaning:**

These results suggest that genetic predisposition to excess body weight is associated with increased risk of mortality among breast cancer survivors and personalized lifestyle recommendations that incorporate genetic predisposition should be considered.

## Teaser

This cohort study investigates whether there is an association between a genetic predisposition to a greater body mass index and mortality risk among US women who survived nonmetastatic breast cancer.

## Introduction

More than 4 million women in the United States are living after a breast cancer diagnosis, and this number is expected to increase due the underlying aging of the population and improvements in diagnosis, treatment, and supportive care.^1^ Concurrently, the rate of obesity, defined as having a body mass index (BMI), calculated as weight in kilograms divided by height in meters squared, of 30 or greater, is increasing in the United States, and it is estimated that 2 of 3 adults will have obesity by 2050.^2^ Overweight (BMI ≥25 to <30) and obesity are prevalent among postmenopausal breast cancer survivors,^3^ which constitutes the majority of breast cancer survivors.^1^ Higher BMI has been associated with poor health outcomes in women diagnosed with breast cancer, including higher risk of mortality.^4,5,6^

BMI is a heritable trait, with heritability estimates ranging between 40% and 70%.^7^ Recent large genome-wide association studies (GWAS) have increased our understanding of the genetic contribution to BMI.^8,9,10^ Polygenic scores (PGSs), which aggregate an individual’s common genetic variants from GWAS findings into a quantitative measure of inherited susceptibility, have been computed for BMI,^11^ and these PGSs have been found to be associated with increased risk of cardiometabolic traits^11,12^ and obesity,^13^ among other conditions.^12,14^ Individuals with high values in this PGS were also more likely to have unhealthy behaviors that lead to obesity.^12^ These studies suggest that PGS may be useful in identifying individuals in need of personalized recommendations to improve health outcomes.

Physical activity is an important component for overall health and for maintaining a healthy weight.^15^ Increased levels of physical activity have been associated with lower risk of mortality.^16,17^ Walking is a common, convenient, and relatively safe form of exercise, which can be recommended to most adults and cancer survivors.^17,18^ However, current recommendations of walking or other forms of physical activity do not take into account personal characteristics or genetic background, although the benefits of exercise may differ by these factors.^13,15,19^

In this analysis, we investigated whether a BMI-PGS was associated with the risk of mortality among breast cancer survivors. Furthermore, we assessed whether the BMI-PGS modified the association between BMI and walking with mortality and whether tailored recommendations should be considered according to their inherited predisposition.

## Methods

### Study Population

This analysis was conducted in the Cancer Prevention Study–II (CPS-II) Nutrition Cohort Study, a prospective cohort of 184 194 cancer-free participants (97 786 women) from 21 US states who returned a survey with demographic information, lifestyle factors, and medical history between 1992 and 1993 and who were subsequently followed up for cancer incidence.^20^ Biennial questionnaires were sent to cohort members starting in 1997 to update exposure information and to ascertain newly diagnosed cancers. Self-reported cancers were verified through abstraction of medical records or linkage with state cancer registries. Information on clinical characteristics was captured from medical records or linkage with US state cancer registries. CPS-II cancer incidence was ascertained from January 1, 1992, through June 30, 2017. Date and the primary underlying cause of death were ascertained via linkage with the National Death Index through December 31, 2020. *International Classification of Diseases* (*ICD*) codes were used to identify specific causes of death, including breast cancer deaths (*ICD*, *Ninth Revision* [*ICD-9*] code 174; ICD, *Tenth Revision *[*ICD-10*] code C50) and cardiovascular disease (CVD) deaths (*ICD-9 *codes 390-398, 402, 404, and 410-429; *ICD-10* codes I00-I09, I11, I13, and I20-I51). Codes related to CVD-specific mortality were defined a priori to primarily represent deaths due to heart disease, including ischemic heart disease, hypertensive heart disease, pulmonary heart disease, and other heart diseases (eg, cardiomyopathy and heart failure), as previously used.^21^ Participants were invited to provide a whole blood sample or buccal cells from mouthwash rinses between 1998 and 2001.^20^ Signed informed consent was received from participants to obtain medical records and from those who provided a blood or buccal sample. The CPS-II Nutrition Cohort study has been approved by the Emory University institutional review board. The study was conducted following the Strengthening the Reporting of Observational Studies in Epidemiology (STROBE) reporting guidelines for cohort studies.

### Genotyping and PGS

Samples from CPS-II participants with breast cancer were genotyped as part of multiple GWAS conducted by consortium groups and have been described elsewhere.^22,23^ More than 90% of our breast cancer samples were genotyped using 3 Illumina chip arrays: the custom iSelect genotyping array as part of the Collaborative Oncological Gene-environment Study (COGS) (iCOGs),^22^ the Infinium OncoArray-500K BeadChip (OncoArray),^23^ or the Infinium Global Screening Array (GSA). Standard quality control of the genome-wide genotyping has been previously described.^22,23^ Samples from 4866 incident breast cancer were genotyped. We excluded 217 participants with genotype information according to the following criteria: discordant phenotype/genotype predicted sex (3 participants), all discordant replicas (53 participants), low genotype rate (<95%; 19 participants), greater than 3-SD heterozygosity (23 participants), or of non-European ancestry or discrepant self-reported race vs genetic ancestry (119 participants).

Single nucleotide variants (SNVs) were excluded if they were missing in more than 5% of samples, their minor allele frequency (MAF) was less 1%, or they were not in Hardy-Weinberg equilibrium (*P* < 1 × 10^−9^). Imputation was performed using the Michigan Imputation Server,^24^ using as reference the Haplotype Reference Consortium (HRC, version r.1.1, GRCh37/hg19), which consists of 64 940 haplotypes of predominantly European ancestry. SNVs with imputation *R*^2^ of greater than 3 and MAF of greater than 0.01 were retained for analysis. Concordant duplicates were merged to obtain maximum coverage. Population substructure analysis was performed using the HapMap3 population.^25^ Analyses were restricted to CPS-II participants of European ancestry given that they were the majority of participants.

The BMI-PGS was calculated using summary statistics from 941 SNVs reported in a meta-analysis of GWAS for BMI that included approximately 700 000 individuals^10^ and the PRSice (version 2.0) software.^26^ Both MAF and reference alleles were compared between the variants reported in Yengo et al^10^ with the variants in the CPS-II data for concordance.

### Assessment of Demographic, Lifestyle Factors, and Treatment Information

Demographic, lifestyle factors, and other personal characteristics were self-reported and obtained from the closest survey before the date of the breast cancer diagnosis. Weight and height were used to compute BMI. BMI was also assessed within 5 years after diagnosis for sensitivity analyses. If multiple postdiagnosis surveys that reported BMI were available, the closest survey to the diagnosis was used. BMI was also retrieved from the 1992 survey, which served as an additional BMI measurement at least 2 years prior to participants’ prediagnosis BMI for a subset of breast cancer survivors for sensitivity analysis. Walking was assessed by asking, “During the past year, what was your average total time per week spent in each of the following activities?” with walking being one of the possible activities in every survey, while the rest of activities varied from survey to survey. The options to report the average time walking per week also varied from survey to survey, with the crudest categories being none or less than 1 hour, 1 to 3 hours, and 4 hours or more. Surveys administered in 1999, 2001, 2005, 2009, and 2011 had similar response options to this question on exercise activities and average time, and the responses to these surveys were used for sensitivity analyses.

First-course treatment for breast cancer was available from linkage to Medicare claims data within 6 months of the cancer diagnosis (available for cases diagnosed 1999-2017) and supplemented with self-reported treatment information. Medicare claims for cancer treatment were identified using established methods and updated with Healthcare Common Procedure Coding System and *ICD* codes.^18^

### Analytical Population

Women who were cancer free before 1992 and who were diagnosed with a first primary breast cancer between 1992 and 2017 were included in the analysis if they had genetic data. We excluded women who did not have a verified BC (239 participants), were diagnosed with distant stage (56 participants), were either premenopausal or younger than 55 years at the time of diagnosis if unknown menopausal status (31 participants), or were genetically related to another case (1 participant). We further excluded women who were 90 years or older at the time of their diagnosis (36 participants) and had underweight (BMI <18.5) or had missing BMI at the time of breast cancer diagnosis (109 participants).

### Statistical Analysis

Breast cancer survivors were followed up from their diagnosis date until their date of death or December 31, 2020, whichever came first. Cumulative all-cause mortality was estimated in tertiles of the BMI-PGS.^27^ Cox proportional hazards regression models were used to calculate hazard ratios (HRs) and 95% CIs for the associations between the BMI-PGS and all-cause mortality, our primary outcome of interest, as well as BC-specific mortality and CVD mortality. Models were adjusted for age at diagnosis (continuous), genotype array (iGOS, OncoArray, GSA, and other), and the first 5 ancestry principal components (PCs) to account for population stratification. Violation of the proportional hazard assumption was assessed by using tests and graphical diagnostics of the slope of the Schoenfeld residuals,^24^ and no significant departures were observed.

Associations between BMI and all-cause, breast cancer–specific mortality, and CVD mortality were also assessed using Cox proportional hazards regression models adjusted for age at diagnosis (continuous), education (high school or less, some college or vocational school, college graduate, graduate school, and unknown), smoking (never, former, current, and unknown), and alcohol (not current drinker, <1 drink/wk, 1-6 drinks/wk, 1 drink/d, ≥2 drinks/d, and unknown). Comorbidities, such as diabetes and hypertension, were not included in the models since they are in the casual pathway. Additionally adjusting for estrogen receptor status, stage, receipt of chemotherapy, receipt of radiation therapy, and receipt of endocrine therapy did not change the results (eTable 1 in Supplement 1) and were not included in the main models. Similar models were run for hours of walking per week as the main exposure and adjusted for the same variables as earlier described and BMI (continuous).

We conducted a formal mediation analysis to investigate whether the association between BMI-PGS and mortality was mediated by BMI using the method proposed by VanderWeele and Vansteelandt via the cmest function in the CMAverse R package^28^ and adjusted for age at diagnosis, genotype array, and the first 5 ancestry PCs. To estimate the risk of all-cause mortality according to hours of walking per week by tertiles of the BMI-PGS, walking was modeled as restricted cubic splines with 3 knots at the 25th, 50th, and 75th percentiles of total hours of walking per week.^29^ Fits and estimates of the Cox proportional hazards regression models with the cubic splines were obtained using the rms R package, version 4.2.2. As in previous models, this analysis was adjusted for age at diagnosis, education, smoking, alcohol, and BMI.

Analyses were conducted using R software version 4.4.1 (R Project for Statistical Computing). All statistical tests were 2-sided, and *P* < .05 were considered statistically significant.

## Results

This analysis included 4177 women diagnosed with nonmetastatic BC. Median (IQR) age at diagnosis was 71.5 (66.3-76.7) years (Table 1). At the time of diagnosis, women were more likely to be never (2204 [52.8%]) or former (1798 [43.0%]) smokers. When characteristics were stratified by tertiles of the BMI-PGS, women in the top tertile, compared with women in the lowest tertile, were more likely to have a BMI of 30 or greater (345 [24.8%] *vs* 172 [12.4%]) (Table 1). Hours of walking were similar across the tertiles of the BMI-PGS. In sensitivity analysis, among 2279 BC survivors with detailed information about walking and nonwalking activities, 2041 (89.6%) reported being active. Walking was one of the forms of exercise for 1976 survivors (96.8%) and the only type of exercise for 529 survivors (25.9%). The distribution of nonwalking activities was similar by tertiles of the BMI-PGS (eFigure 1 in Supplement 1). There were no major differences in clinical characteristics or treatment received according to the BMI-PGS, with most women being diagnosed with localized (2628 [62.9%] (Table 1) and estrogen receptor–positive (2753 [65.9%]) disease.

**Table 1.** Sample Characteristics

Characteristic at diagnosis	Participants, No. (%)			
	Overall (n = 4177)	Tertiles of the BMI-PGS		
		1 (n = 1392)	2 (n = 1392)	3 (n = 1393)
Age, y				
Median (IQR)	71.5 (66.3-76.7)	71.7 (66.3-76.7)	71.4 (66.3-76.4)	71.4 (66.3-77.1)
<60	260 (6.2)	87 (6.3)	86 (6.2)	87 (6.2)
60 to <65	581 (13.9)	194 (13.9)	195 (14.0)	192 (13.8)
65 to <70	927 (22.2)	307 (22.1)	320 (23.0)	300 (21.5)
70 to <75	1034 (24.8)	332 (23.9)	353 (25.4)	349 (25.1)
≥75	1375 (32.9)	472 (33.9)	438 (31.5)	465 (33.4)
Education				
High school or less	1301 (31.1)	400 (28.7)	449 (32.3)	452 (32.4)
Some college or vocational school	1288 (30.8)	459 (33.0)	396 (28.4)	433 (31.1)
College graduate	941 (22.5)	309 (22.2)	324 (23.3)	308 (22.1)
Graduate school	624 (14.9)	211 (15.2)	216 (15.5)	197 (14.1)
Unknown	23 (0.6)	13 (0.9)	7 (0.5)	3 (0.2)
Insurance				
Employer insurance	963 (23.1)	313 (22.5)	339 (24.4)	311 (22.3)
Medicare or Medicaid	2802 (67.1)	959 (68.9)	918 (65.9)	925 (66.4)
Self-bought insurance	252 (6.0)	70 (5.0)	79 (5.7)	103 (7.4)
Military or other insurance	97 (2.3)	27 (1.9)	33 (2.4)	37 (2.7)
No insurance	12 (0.3)	5 (0.4)	6 (0.4)	1 (0.1)
Unknown	51 (1.2)	18 (1.3)	17 (1.2)	16 (1.1)
BMI				
<25	2059 (49.3)	796 (57.2)	688 (49.4)	575 (41.3)
25 to <30	1364 (32.7)	424 (30.5)	467 (33.5)	473 (34.0)
≥30	754 (18.1)	172 (12.4)	237 (17.0)	345 (24.8)
Smoking status				
Never	2204 (52.8)	738 (53.0)	737 (52.9)	729 (52.3)
Former	1798 (43.0)	602 (43.2)	594 (42.7)	602 (43.2)
Current	165 (4.0)	46 (3.3)	58 (4.2)	61 (4.4)
Unknown	10 (0.2)	6 (0.4)	3 (0.2)	1 (0.1)
Alcohol use				
Nondrinker	1894 (45.3)	606 (43.5)	631 (45.3)	657 (47.2)
<1 drink/wk	534 (12.8)	175 (12.6)	183 (13.1)	176 (12.6)
1-6 drinks/wk	1086 (26.0)	391 (28.1)	349 (25.1)	346 (24.8)
1 drink/d	406 (9.7)	143 (10.3)	132 (9.5)	131 (9.4)
≥2 drinks/d	226 (5.4)	69 (5.0)	86 (6.2)	71 (5.1)
Missing	31 (0.7)	8 (0.6)	11 (0.8)	12 (0.9)
Walking, h/wk				
None or <1	808 (19.3)	240 (17.2)	261 (18.8)	307 (22.0)
1-3	2019 (48.3)	667 (47.9)	691 (49.6)	661 (47.5)
≥4	1307 (31.3)	472 (33.9)	430 (30.9)	405 (29.1)
Missing	43 (1.0)	13 (0.9)	10 (0.7)	20 (1.4)
CVD status^a^				
No	3643 (87.2)	1227 (88.1)	1197 (86.0)	1219 (87.5)
Yes	370 (8.9)	110 (7.9)	134 (9.6)	126 (9.0)
Missing	164 (3.9)	55 (4.0)	61 (4.4)	48 (3.4)
Diabetes status				
No	3503 (83.9)	1191 (85.6)	1165 (83.7)	1147 (82.3)
Yes	501 (12.0)	143 (10.3)	165 (11.9)	193 (13.9)
Missing	173 (4.1)	58 (4.2)	62 (4.5)	53 (3.8)
Hypertension status				
No	2090 (50.0)	731 (52.5)	698 (50.1)	661 (47.5)
Yes	2015 (48.2)	632 (45.4)	673 (48.3)	710 (51.0)
Missing	72 (1.7)	29 (2.1)	21 (1.5)	22 (1.6)
Stage				
In situ	760 (18.2)	240 (17.2)	275 (19.8)	245 (17.6)
Localized	2628 (62.9)	877 (63.0)	845 (60.7)	906 (65.0)
Regional	733 (17.5)	250 (18.0)	254 (18.2)	229 (16.4)
Missing	56 (1.3)	25 (1.8)	18 (1.3)	13 (0.9)
Estrogen receptor status				
Negative	461 (11.0)	156 (11.2)	155 (11.1)	150 (10.8)
Positive	2753 (65.9)	914 (65.7)	909 (65.3)	930 (66.8)
Unknown	963 (23.1)	322 (23.1)	328 (23.6)	313 (22.5)
Progesterone receptor status				
Negative	838 (20.1)	286 (20.5)	284 (20.4)	268 (19.2)
Positive	2275 (54.5)	748 (53.7)	748 (53.7)	779 (55.9)
Unknown	1064 (25.5)	358 (25.7)	360 (25.9)	346 (24.8)
*ERBB2* status				
Negative	1298 (31.1)	425 (30.5)	439 (31.5)	434 (31.2)
Positive	224 (5.3)	85 (6.1)	54 (3.9)	85 (6.1)
Unknown	2655 (63.6)	882 (63.4)	899 (64.6)	874 (62.7)
Breast surgery				
No	24 (0.6)	7 (0.5)	8 (0.6)	9 (0.6)
Yes	3658 (87.6)	1223 (87.9)	1213 (87.1)	1222 (87.7)
Unknown	495 (11.9)	162 (11.7)	171 (12.3)	162 (11.6)
Radiation therapy				
No	1489 (35.6)	487 (35.0)	502 (36.1)	500 (35.9)
Yes	2478 (59.3)	843 (60.6)	812 (58.3)	823 (59.1)
Unknown	210 (5.0)	62 (4.5)	78 (5.6)	70 (5.0)
Chemotherapy				
No	3081 (73.8)	1022 (73.4)	1011 (72.6)	1048 (75.2)
Yes	886 (21.2)	308 (22.1)	303 (21.8)	275 (19.7)
Unknown	210 (5.0)	62 (4.5)	78 (5.6)	70 (5.0)
Endocrine therapy				
No	1178 (28.2)	393 (28.2)	401 (28.8)	384 (27.6)
Yes	2529 (60.5)	845 (60.7)	836 (60.1)	848 (60.9)
Unknown	470 (11.3)	154 (11.1)	155 (11.1)	161 (11.6)
Vital status				
Alive	2063 (49.4)	712 (51.1)	697 (50.1)	654 (46.9)
Dead	2114 (50.6)	680 (48.9)	695 (49.9)	739 (53.1)
Primary cause of death: breast cancer	355 (16.8)	108 (15.9)	130 (18.7)	117 (15.8)
Primary cause of death: CVD	400 (18.9)	133 (19.6)	130 (18.7)	137 (18.5)
Follow-up time from diagnosis to death, median (IQR), y	14.5 (9.7-19.7)	15.0 (10.0-20.2)	14.4 (10.1-19.6)	14.1 (9.2-19.4)

Abbreviations: BMI, body mass index (calculated as weight in kilograms divided by height in meters squared); BMI-PGS, polygenic score for body mass index; CVD, cardiovascular disease.

^a^ CVD status includes heart disease, coronary artery disease, bypass surgery, angina pectoris, heart attack or myocardial infarction, and/or carotid surgery, stroke, and transient ischemic attack.

Given that cancer and its treatments can influence weight, the association between BMI reported prior to the cancer diagnosis (median [IQR] time from assessment to diagnosis, 1.2 [0.6-1.9] years) and within 5 years after diagnosis (median [IQR] time from diagnosis to assessment, 1.2 [0.6-1.8] years) was investigated. We found that BMI was highly correlated between these 2 time points (Pearson correlation, 0.93), and more than 83.6% of the women had the same categorical classification of BMI (eFigure 2A in Supplement 1). In addition, among 3088 women (73.9%) who had an additional BMI measurement at least 2 years prior to their prediagnosis BMI (median [IQR] time between measurements, 9.9 [7.0-14.4] years), BMI at this time was also highly correlated with the BMI reported prior to cancer diagnosis (Pearson correlation, 0.87) and 75.9% had the same categorical classification of BMI (eFigure 2B in Supplement 1).

During a median (IQR) follow-up of 14.5 (9.7-19.7) years (Table 1), 2114 breast cancer survivors (50.6%) died. The primary cause of death for 355 (16.8%) was BC, and for 400 (18.9%), it was CVD. Higher BMI was associated with increased risk of all-cause mortality (HR per 5 units of BMI, 1.09; 95% CI, 1.04-1.14) (eTable 1 in Supplement 1), and greater time walking was associated with reduced risk of all-cause mortality (≥4 h/wk vs <1 h/wk: HR, 0.74; 95% CI, 0.66-0.84) among breast cancer survivors.

**Figure 1.**
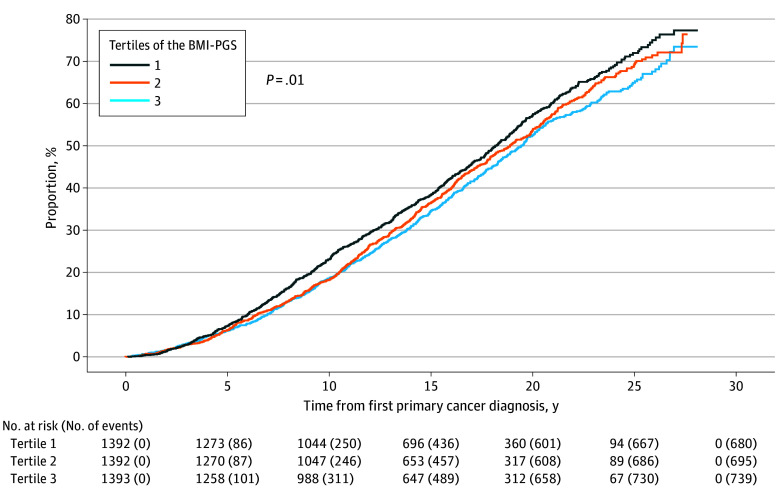
Cumulative All-Cause Mortality by Tertile of the Polygenic Score for Body Mass Index (BMI-PGS)

Higer BMI-PGS was associated with increased risk of all-cause mortality (Figure 1). The 10-year cumulative all-cause mortality for BC survivors who were in the highest tertile of the BMI-PGS was 23.1% (95% CI, 20.9%-25.4%), and it was 18.6% (95% CI, 16.5%-20.7%) for BC survivors who were in the lowest tertile of the BMI-PGS. An increase of 1 SD in the BMI-PGS was associated with a 7% increased risk of all-cause mortality (HR, 1.07; 95% CI, 1.02-1.12) (Table 2). Compared with breast cancer survivors in the lowest tertile of the BMI-PGS, those in the highest tertile had a 15% increased risk of all-cause mortality (HR, 1.15; 95% CI, 1.04-1.28). The HRs for breast cancer–specific and CVD-specific mortality for an increase of 1 SD in the BMI-PGS were both 1.06, although results did not reach statistical significance (breast cancer–specific mortality: HR, 1.06; 95% CI, 0.96-1.18; CVD-specific mortality: HR, 1.06; 95% CI, 0.97-1.18 (eTable 2 in Supplement 1).

**Table 2.** Associations Between the BMI-PGS and All-Cause Mortality

BMI-PGS	Participants, No. (%)		HR (95% CI)^a^
	BC survivors (n = 4177)	Deaths (n = 2114)	
Continuous, per 1 SD	4177 (100)	2114 (100)	1.07 (1.02-1.12)
Tertiles			
1	1392 (33.3)	680 (32.2)	1 [Reference]
2	1392 (33.3)	695 (32.9)	1.08 (0.97-1.20)
3	1393 (33.3)	739 (35.0)	1.15 (1.04-1.28)

Abbreviations: BC, breast cancer; BMI-PGS, polygenic score for body mass index; HR, hazard ratio; PGS, polygenic score.

^a^ Adjusted for age (continuous), first 5 principal components for population stratification, and genotyping chip array.

Given that the BMI-PGS was significantly associated with BMI (β = 0.17 [95% CI, 0.15-0.20] per 1-SD change in the BMI-PGS), we explored whether the observed increased risk of all-cause mortality associated with the BMI-PGS could be explained by BMI (eFigure 3 in Supplement 1). We found that 32.7% (95% CI, 16.8%-74.4%) of the association was mediated by BMI.

While BMI and hours of walking per week were associated with all-cause mortality, we examined whether these associations differed across tertiles of the BMI-PGS (Table 3). Regardless of the tertile of the BMI-PGS, having a BMI of 30 or greater was associated with approximately 30% increased risk of all-cause mortality compared with having a BMI less than 25, and walking for 4 hours or more a week with approximately 25% decreased risk of all-cause mortality compared with walking less than 1 hour a week.

**Table 3.** Associations Between Modifiable Factors and All-Cause Mortality, Stratified by Tertiles of the BMI-PGS

Modifiable factor	Tertiles of the BMI-PGS											
	1				2				3			
	No. (%)		HR (95% CI)^a^	*P* value for trend	No. (%)		HR (95% CI)^a^	*P* value for trend	No. (%)		HR (95% CI)^a^	*P* value for trend
	BC survivors (n = 1392)	Deaths (n = 680)			BC survivors (n = 1392)	Deaths (n = 695)			BC survivors (n = 1393)	Deaths (n = 739)		
BMI												
Continuous, per 5 units	1392 (100)	680 (100)	1.14 (1.05-1.25)	NA	1392 (100)	695 (100)	1.11 (1.01-1.20)	NA	1393 (100)	739 (100)	1.14 (1.06-1.23)	NA
Categorical												
<25	796 (57.2)	386 (56.8)	1 [Reference]	.03	688 (49.4)	330 (47.5)	1 [Reference]	.03	575 (41.3)	306 (41.4)	1 [Reference]	.01
≥25 to <30	424 (30.5)	206 (30.3)	1.05 (0.88-1.25)		467 (33.5)	235 (33.8)	1.10 (0.93-1.30)		473 (34.0)	242 (32.7)	1.00 (0.84-1.18)	
≥30	172 (12.4)	88 (12.9)	1.37 (1.08-1.73)		237 (17.0)	130 (18.7)	1.26 (1.02-1.55)		345 (24.8)	191 (25.8)	1.31 (1.09-1.58)	
Walking, h/wk												
None or <1	240 (17.2)	135 (19.9)	1 [Reference]	.04	261 (18.8)	149 (21.4)	1 [Reference]	.049	307 (22.0)	190 (25.7)	1 [Reference]	<.001
1-3	667 (47.9)	324 (47.6)	0.73 (0.59-0.90)		691 (49.6)	345 (49.6)	0.77 (0.63-0.95)		661 (47.5)	361 (48.8)	0.84 (0.70-1.01)	
≥4	472 (33.9)	214 (31.5)	0.75 (0.60-0.94)		430 (30.9)	196 (28.2)	0.78 (0.63-0.98)		405 (29.1)	176 (23.8)	0.68 (0.55-0.85)	

Abbreviations: BC, breast cancer; BMI, body mass index (calculated as weight in kilograms divided by height in meters squared); BMI-PGS, polygenic score for body mass index; HR, hazard ratio; PGS, polygenic score.

^a^ Models for BMI as the main exposure were adjusted age at diagnosis (continuous), education (high school or less, some college or vocational school, college graduate, graduate school, unknown), smoking (never, former, current, unknown), and alcohol (not current drinker, <1 drink/wk, 1-6 drinks/wk, 1 drink/d, ≥2 drinks/d, unknown). Models with walking as the main exposures were adjusted for all the previously mentioned variables and BMI (continuous).

**Figure 2.**
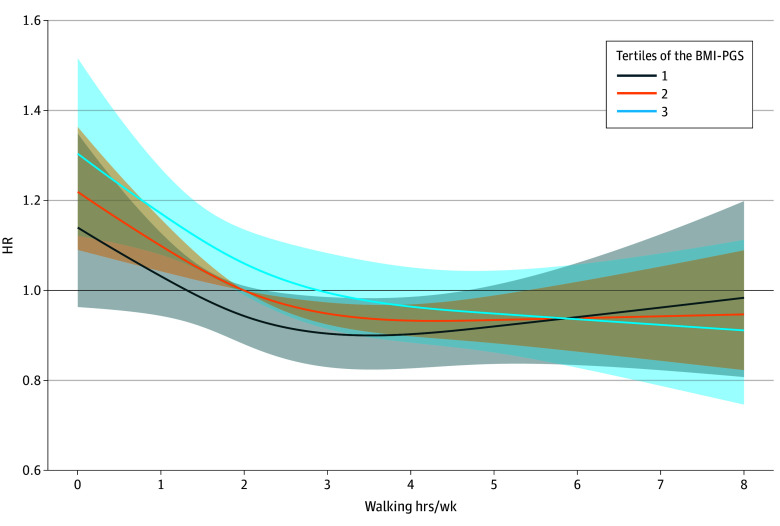
Risk of All-Cause Mortality According to Hours of Walking Per Week by Tertiles of the Polygenic Score for Body Mass Index (BMI-PGS) The hazard ratio (HR) for all-cause mortality was modeled according to hours of walking per week and tertiles of the polygenic score for body mass index. Shaded regions represent 95% CIs. The model was adjusted for age, education, smoking, alcohol, body mass index, and an interaction term between the polygenic risk score and hours of walking per week.

Since higher BMI-PGS was associated with increased risk of all-cause mortality, we estimated the hours per week needed to reduce the risk of mortality for individuals in each tertile of the BMI-PGS (Figure 2). We found that breast cancer survivors with a BMI-PGS in the lowest tertile needed approximately 1.3 hours per week to reach a hazard ratio of 1.00 (HR, 1.00; 95% CI, 0.93-1.08). Similarly, breast cancer survivors with a BMI-PGS in the highest tertile needed 2.9 hours of walking per week to reach a hazard ratio of 1.00 (HR, 1.00; 95% CI, 0.92-1.09).

## Discussion

In this cohort study of breast cancer survivors, those who had a higher genetic predisposition to excess body weight had an increased risk of all-cause mortality, highlighting the importance of implementing targeted prevention strategies to mitigate their genetic risk. Specifically, women with breast cancer whose BMI-PGS was in the highest tertile had a 15% increased risk of all-cause mortality compared with women whose BMI-PGS was in the lowest tertile. We observed that BMI and walking, both modifiable risk factors, had similar associations with the risk of all-cause mortality, regardless of the excess body weight genetic predisposition, suggesting that any breast cancer survivor benefits equally from greater hours of walking and experiences the same harms from higher BMI. However, we found that breast cancer survivors with a BMI-PGS in the highest tertile needed to walk approximately an additional 14 minutes per day to be at a similar risk as breast cancer survivors in the lowest tertile of the BMI-PGS.

Previous studies have shown that BMI-related PGSs have been associated with obesity and several diseases and conditions.^12,14^ Here we observed that the BMI-PGS was associated with all-cause mortality in breast cancer survivors. We explored whether this association was explained by BMI and found that some of the associations of the BMI-PGS with mortality were independent of BMI. While the mechanisms underlying the associations between BMI-PGS and mortality are unclear, Yengo et al^10^ found 138 different genes for which expression could mediate the associations between the SNVs and BMI. These genes have pleiotropic effects, with most of them being involved in neurogenesis and the development of the central nervous system.^10^ Moreover, a recent study found that a PGS for BMI was associated with a broad range of diseases, including life-threatening conditions of the genitourinary (eg, chronic kidney failure) and respiratory (eg, respiratory failure, asthma) systems as well as conditions associated with substantial morbidity in the musculoskeletal and dermatologic systems.^14^

In our study of older breast cancer survivors, BMI was fairly consistent before and after the cancer diagnosis. This highlights the unique challenges that this older population faces to change their weight. While it is known that obesity has a heritable component, genetic predisposition is not deterministic of obesity.^30^ In fact, in our study, 12.4% of women in the lowest tertile of the BMI-PGS had a BMI of 30 or greater, and 41.3% of the women in the highest tertile of the BMI-PGS had a BMI less than 25, suggesting the importance of other factors, including healthy lifestyle choices. Information on PGS has the potential to encourage risk-reduction behaviors and lifestyle changes^31,32^ and provide targeted recommendations.^33^

It is well established that greater time spent walking has been associated with reduced mortality,^29^ including in older women.^16,17^ In our study of older breast cancer survivors, walking was a common form of exercise. However, walking for 150 minutes per week, which may satisfy the recommendations of moderate-intensity aerobic activity per week, may not benefit all women the same. A recent study found that individuals at high genetic risk of obesity needed higher daily step counts to reduce the risk of obesity than those at moderate or low genetic risk.^13^ We also estimated the amount of walking that was needed to offset their genetic predisposition to excess body weight and found that those with a high predisposition needed to walk approximately 1.7 hours per week more than breast cancer survivors with a low predisposition. These results underscore the importance of making personalized recommendations that include genetic background.

### Limitations

The findings from this analysis should be considered with certain limitations. Our population was relatively healthy, with lower rates of obesity compared with current rates in the general population,^2^ and therefore, we may have underestimated the risk of mortality. Most GWAS of BMI have included participants of European ancestries. It is unclear whether the same genetic variants could be applicable to other ancestries and therefore whether our results are generalizable to other populations.^34^ Larger sample sizes and more refined collection of walking information may be needed to more precisely estimate the amount of walking needed to mitigate the genetic background according the BMI-PGS. We had limited data on other nonwalking activities, and future studies should consider including them in the analysis.

## Conclusions

BC survivors who were genetically predisposed to having a higher BMI were at increased risk of all-cause mortality. The genetic predisposition did not modify the associations between BMI or hours of walking per week and all-cause mortality, and therefore the benefits and harms of these modifiable behaviors are similar regardless of the genetic background. However, given the increased risk associated with genetic predisposition, targeted recommendations that include genetic information may be beneficial for reducing the risk of mortality.

## Notes

Supplement 1. **eFigure 1.** Distribution of Hours per Week Spent on Nonwalking Activities According to the BMI-PGS **eFigure 2.** Association Between BMI at Different Times During the Study Period **eTable 1.** Associations Between BMI and Hours of Walking per Week With All-Cause, BC-, and CVD-Specific Mortality **eTable 2.** Associations of the BMI-PGS With BC-Specific and CVD-Specific Mortality **eFigure 3.** Causal Diagram of the Relationship Between the BMI-PGS, BMI, and All-Cause Mortality

Supplement 2.
Data Sharing Statement

## References

[1] Tonorezos E, Devasia T, Mariotto AB, . Prevalence of cancer survivors in the United States. J Natl Cancer Inst. 2024;116(11):1784-1790. doi:10.1093/jnci/djae135 39002121 PMC11542986

[2] Ng M, Dai X, Cogen RM, ; GBD 2021 US Obesity Forecasting Collaborators. National-level and state-level prevalence of overweight and obesity among children, adolescents, and adults in the USA, 1990-2021, and forecasts up to 2050. Lancet. 2024;404(10469):2278-2298. doi:10.1016/S0140-6736(24)01548-4 39551059 PMC11694015

[3] Lauby-Secretan B, Scoccianti C, Loomis D, Grosse Y, Bianchini F, Straif K; International Agency for Research on Cancer Handbook Working Group. Body fatness and cancer—viewpoint of the IARC Working Group. N Engl J Med. 2016;375(8):794-798. doi:10.1056/NEJMsr1606602 27557308 PMC6754861

[4] Chan DSM, Vieira AR, Aune D, . Body mass index and survival in women with breast cancer-systematic literature review and meta-analysis of 82 follow-up studies. Ann Oncol. 2014;25(10):1901-1914. doi:10.1093/annonc/mdu042 24769692 PMC4176449

[5] Greenlee H, Unger JM, LeBlanc M, Ramsey S, Hershman DL. Association between body mass index and cancer survival in a pooled analysis of 22 clinical trials. Cancer Epidemiol Biomarkers Prev. 2017;26(1):21-29. doi:10.1158/1055-9965.EPI-15-1336 27986655 PMC5370550

[6] Maliniak ML, Patel AV, McCullough ML, . Obesity, physical activity, and breast cancer survival among older breast cancer survivors in the Cancer Prevention Study-II Nutrition Cohort. Breast Cancer Res Treat. 2018;167(1):133-145. doi:10.1007/s10549-017-4470-7 28856470

[7] Elks CE, den Hoed M, Zhao JH, . Variability in the heritability of body mass index: a systematic review and meta-regression. Front Endocrinol (Lausanne). 2012;3:29. doi:10.3389/fendo.2012.00029 22645519 PMC3355836

[8] Speakman JR, Loos RJF, O’Rahilly S, Hirschhorn JN, Allison DB. GWAS for BMI: a treasure trove of fundamental insights into the genetic basis of obesity. Int J Obes (Lond). 2018;42(8):1524-1531. doi:10.1038/s41366-018-0147-5 29980761 PMC6115287

[9] Locke AE, Kahali B, Berndt SI, ; LifeLines Cohort Study; ADIPOGen Consortium; AGEN-BMI Working Group; CARDIOGRAMplusC4D Consortium; CKDGen Consortium; GLGC; ICBP; MAGIC Investigators; MuTHER Consortium; MIGen Consortium; PAGE Consortium; ReproGen Consortium; GENIE Consortium; International Endogene Consortium. Genetic studies of body mass index yield new insights for obesity biology. Nature. 2015;518(7538):197-206. doi:10.1038/nature14177 25673413 PMC4382211

[10] Yengo L, Sidorenko J, Kemper KE, ; GIANT Consortium. Meta-analysis of genome-wide association studies for height and body mass index in ∼700000 individuals of European ancestry. Hum Mol Genet. 2018;27(20):3641-3649. doi:10.1093/hmg/ddy271 30124842 PMC6488973

[11] Khera AV, Chaffin M, Wade KH, . Polygenic prediction of weight and obesity trajectories from birth to adulthood. Cell. 2019;177(3):587-596.e9. doi:10.1016/j.cell.2019.03.028 31002795 PMC6661115

[12] Jansen PR, Vos N, van Uhm J, . The utility of obesity polygenic risk scores from research to clinical practice: a review. Obes Rev. 2024;25(11):e13810. doi:10.1111/obr.13810 39075585

[13] Brittain EL, Han L, Annis J, . Physical activity and incident obesity across the spectrum of genetic risk for obesity. JAMA Netw Open. 2024;7(3):e243821. doi:10.1001/jamanetworkopen.2024.3821 38536175 PMC10973894

[14] Huang J, Huffman JE, Huang Y, ; VA Million Veteran Program. Genomics and phenomics of body mass index reveals a complex disease network. Nat Commun. 2022;13(1):7973. doi:10.1038/s41467-022-35553-2 36581621 PMC9798356

[15] Donnelly JE, Blair SN, Jakicic JM, Manore MM, Rankin JW, Smith BK; American College of Sports Medicine. American College of Sports Medicine position stand: appropriate physical activity intervention strategies for weight loss and prevention of weight regain for adults. Med Sci Sports Exerc. 2009;41(2):459-471. doi:10.1249/MSS.0b013e3181949333 19127177

[16] Patel AV, Hildebrand JS, Leach CR, . Walking in relation to mortality in a large prospective cohort of older U.S. adults. Am J Prev Med. 2018;54(1):10-19. doi:10.1016/j.amepre.2017.08.019 29056372

[17] Lee IM, Shiroma EJ, Kamada M, Bassett DR, Matthews CE, Buring JE. Association of step volume and intensity with all-cause mortality in older women. JAMA Intern Med. 2019;179(8):1105-1112. doi:10.1001/jamainternmed.2019.0899 31141585 PMC6547157

[18] Campbell KL, Winters-Stone KM, Wiskemann J, . Exercise guidelines for cancer survivors: consensus statement from International Multidisciplinary Roundtable. Med Sci Sports Exerc. 2019;51(11):2375-2390. doi:10.1249/MSS.0000000000002116 31626055 PMC8576825

[19] Ji H, Gulati M, Huang TY, . Sex differences in association of physical activity with all-cause and cardiovascular mortality. J Am Coll Cardiol. 2024;83(8):783-793. doi:10.1016/j.jacc.2023.12.019 38383092 PMC10984219

[20] Calle EE, Rodriguez C, Jacobs EJ, . The American Cancer Society Cancer Prevention Study II Nutrition Cohort: rationale, study design, and baseline characteristics. Cancer. 2002;94(9):2490-2501. doi:10.1002/cncr.101970 12015775

[21] Ramin C, Schaeffer ML, Zheng Z, . All-cause and cardiovascular disease mortality among breast cancer survivors in CLUE II, a long-standing community-based cohort. J Natl Cancer Inst. 2021;113(2):137-145. doi:10.1093/jnci/djaa096 32634223 PMC7850550

[22] Michailidou K, Hall P, Gonzalez-Neira A, . Large-scale genotyping identifies 41 new loci associated with breast cancer risk. Nat Genet. 2013;45(4):353-61, 361e1-2. doi:10.1038/ng.2563 PMC3771688 23535729

[23] Michailidou K, Lindström S, Dennis J, ; NBCS Collaborators; ABCTB Investigators; ConFab/AOCS Investigators. Association analysis identifies 65 new breast cancer risk loci. Nature. 2017;551(7678):92-94. doi:10.1038/nature24284 29059683 PMC5798588

[24] Das S, Forer L, Schönherr S, . Next-generation genotype imputation service and methods. Nat Genet. 2016;48(10):1284-1287. doi:10.1038/ng.3656 27571263 PMC5157836

[25] Broad Institute. HapMap 3. Accessed December 1, 2025. https://www.broadinstitute.org/medical-and-population-genetics/hapmap-3

[26] Choi SW, O’Reilly PF. PRSice-2: polygenic risk score software for biobank-scale data. Gigascience. 2019;8(7):giz082. doi:10.1093/gigascience/giz082 31307061 PMC6629542

[27] Gray RJ. A class of *K*-sample tests for comparing the cumulative incidence of a competing risk. *Ann Stat*. 1988;16(3):1141-1154.

[28] Shi B, Choirat C, Coull BA, VanderWeele TJ, Valeri L. CMAverse: a suite of functions for reproducible causal mediation analyses. Epidemiology. 2021;32(5):e20-e22. doi:10.1097/EDE.0000000000001378 34028370

[29] Paluch AE, Bajpai S, Bassett DR, ; Steps for Health Collaborative. Daily steps and all-cause mortality: a meta-analysis of 15 international cohorts. Lancet Public Health. 2022;7(3):e219-e228. doi:10.1016/S2468-2667(21)00302-9 35247352 PMC9289978

[30] Murthy VL, Xia R, Baldridge AS, . Polygenic risk, fitness, and obesity in the Coronary Artery Risk Development in Young Adults (CARDIA) study. JAMA Cardiol. 2020;5(3):40-48. doi:10.1001/jamacardio.2019.5220 31913407 PMC6990863

[31] Adeyemo A, Balaconis MK, Darnes DR, ; Polygenic Risk Score Task Force of the International Common Disease Alliance. Responsible use of polygenic risk scores in the clinic: potential benefits, risks and gaps. Nat Med. 2021;27(11):1876-1884. doi:10.1038/s41591-021-01549-6 34782789

[32] Widén E, Junna N, Ruotsalainen S, . How communicating polygenic and clinical risk for atherosclerotic cardiovascular disease impacts health behavior: an observational follow-up study. Circ Genom Precis Med. 2022;15(2):e003459. doi:10.1161/CIRCGEN.121.003459 35130028

[33] Yurkovich JT, Evans SJ, Rappaport N, . The transition from genomics to phenomics in personalized population health. Nat Rev Genet. 2024;25(4):286-302. doi:10.1038/s41576-023-00674-x 38093095

[34] Martin AR, Kanai M, Kamatani Y, Okada Y, Neale BM, Daly MJ. Clinical use of current polygenic risk scores may exacerbate health disparities. Nat Genet. 2019;51(4):584-591. doi:10.1038/s41588-019-0379-x 30926966 PMC6563838

