# Supplementary material for: Genetic Predisposition to Excess Body Weight and Survival in Women Diagnosed With Breast Cancer
Source: JAMA Netw Open. 2026 Jan 13;9(1):e2553687. doi: 10.1001/jamanetworkopen.2025.53687 (PMC12801084; doi:10.1001/jamanetworkopen.2025.53687)
Supplement: Supplement 1. — eFigure 1. Distribution of Hours per Week Spent on Nonwalking Activities According to the BMI-PGS eFigure 2. Association Between BMI at Different Times During the Study Period eTable 1. Associations Between BMI and Hours of Walking per Week With All-Cause, BC-, and CVD-Specific Mortality eTable 2. Associations of the BMI-PGS With BC-Specific and CVD-Specific Mortality eFigure 3. Causal Diagram of the Relationship Between the BMI-PGS, BMI, and All-Cause Mortality [file jamanetwopen-e2553687-s001.pdf]

Supplemental Online Content

Bodelon C, Landry M, Lori A, et al. Genetic predisposition to excess body weight and survival in women diagnosed with breast cancer. *JAMA Netw Open.* 2026;9(1):e2553687. doi:10.1001/jamanetworkopen.2025.53687

eFigure 1. Distribution of Hours per Week Spent on Nonwalking Activities According to the BMI-PGS

eFigure 2. Association Between BMI at Different Times During the Study Period

eTable 1. Associations Between BMI and Hours of Walking per Week With All-Cause, BC-, and CVD-Specific Mortality

eTable 2. Associations of the BMI-PGS With BC-Specific and CVD-Specific Mortality

eFigure 3. Causal Diagram of the Relationship Between the BMI-PGS, BMI, and All-Cause Mortality

This supplemental material has been provided by the authors to give readers additional information about their work.

© 2026 Bodelon C et al. *JAMA Network Open*.

eFigure 1. Distribution of Hours per Week Spent on Nonwalking Activities According to the BMI-PGS

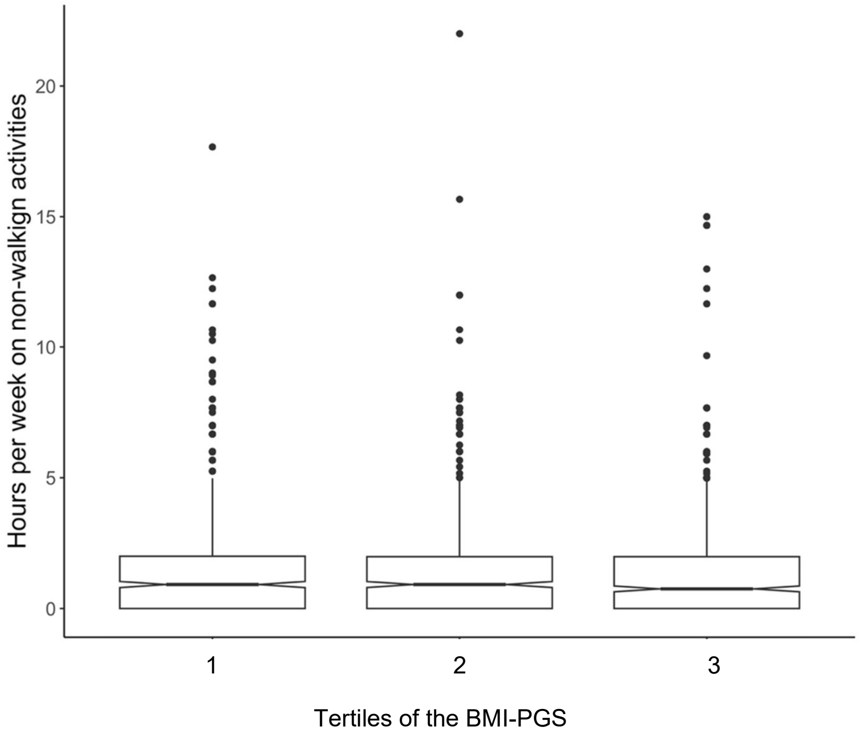

© 2026 Bodelon C et al. *JAMA Network Open*.

eFigure 2. Association Between BMI at Different Times During the Study Period
A. Change of body mass index (BMI) before and after breast cancer diagnosis. Median years from BMI measurements to diagnosis was 1.2 years (interquartile range: 0.6-1.9) and from diagnosis to BMI measurement was 1.2 years (interquartile range: 0.6-1.8). **B.** Change of BMI closest to diagnosis and at least 2 years before diagnosis among 3,088 (73.9%) of women who had both measurements. Median time between measurements: 9.9 years (interquartile range: 7.0-14.4).

A. **B.**

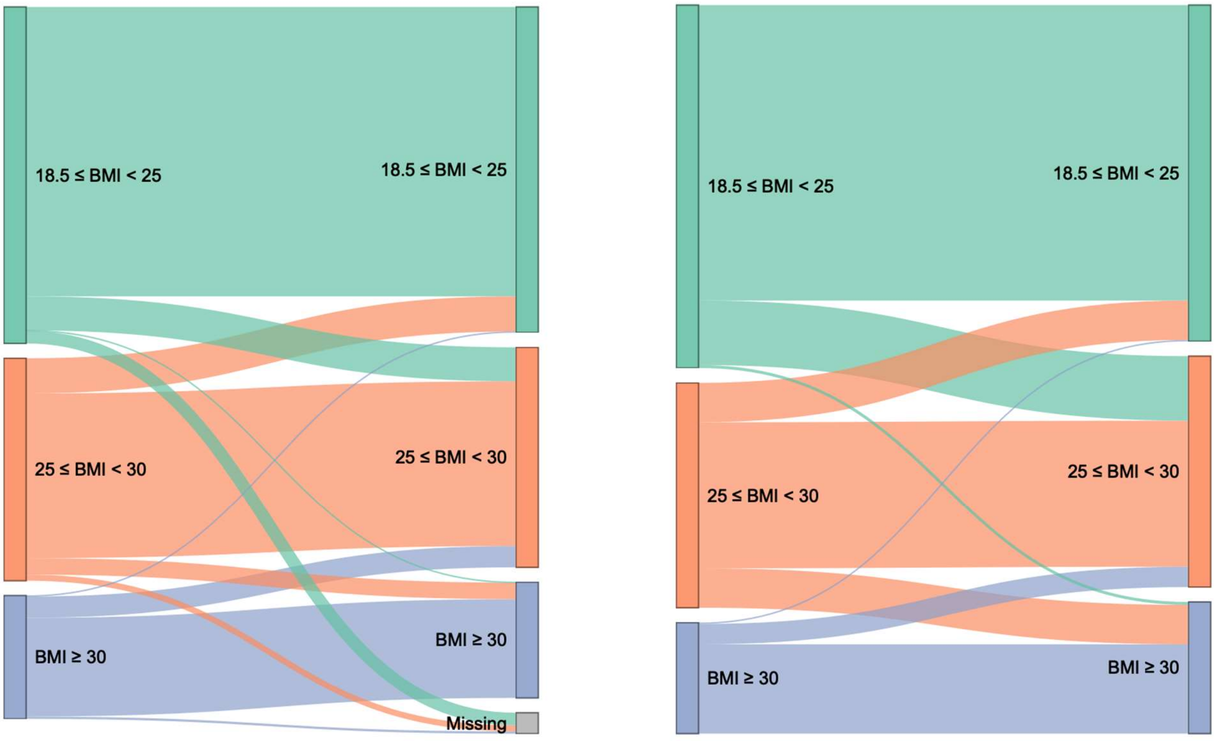

© 2026 Bodelon C et al. *JAMA Network Open*.

eTable 1. Associations Between BMI and Hours of Walking per Week With All-Cause, BC-, and CVD-Specific Mortality

Modifiable factor	BC survivors (N=4,177) N (%)	Deaths N (%)	HR (95% CI)[a]	HR (95% CI)[b]
**All cause-mortality**		**N=2,114**		
**BMI**				
Continuous (per 5 kg/m^2)	4,177 (100)	2,114 (100)	1.14 (1.09-1.19)	1.13 (1.08-1.19)
Categorical (kg/m^2)				
BMI<25	2,059 (48.0)	1,022 (46.7)	1.00 (Ref.)	1.00 (Ref.)
25≤BMI<30	1,364 (31.8)	683 (31.2)	1.05 (0.96-1.16)	1.04 (0.94-1.15)
BMI≥30	754 (18.1)	409 (19.3)	1.33 (1.18-1.50)	1.31 (1.16-1.48)
P-trend			<0.001	<0.001
**Walking (hours/week)**				
Categorical (hours/week)				
<1	808 (19.3)	474 (22.4)	1.00 (Ref.)	1.00 (Ref.)
1-≤3	2,019 (48.4)	1,030 (48.7)	0.78 (0.70-0.88)	0.80 (0.71-0.89)
≥4	1,307 (31.3)	586 (27.7)	0.73 (0.65-0.83)	0.74 (0.65-0.84)
P-trend			<0.001	<0.001
**BC specific-mortality**		**N=355**		
**BMI**				
Continuous (per 5 kg/m^2)	4,177 (100)	355 (100)	1.21 (1.09-1.34)	1.19 (1.08-1.32)
Categorical (kg/m^2)				
BMI<25	2,059 (48.0)	156 (43.9)	1.00 (Ref.)	1.00 (Ref.)
25≤BMI<30	1,364 (31.8)	120 (33.8)	1.19 (0.94-1.51)	1.10 (0.86-1.40)
BMI≥30	754 (18.1)	79 (22.3)	1.45 (1.10-1.91)	1.38 (1.05-1.83)
P-trend			0.008	0.029
**Walking (hours/week)**				
Categorical (hours/week)				
<1	808 (19.3)	78 (22.0)	1.00 (Ref.)	1.00 (Ref.)
1-≤3	2,019 (48.4)	167 (47.0)	0.77 (0.58-1.01)	0.76 (0.58-1.00)
≥4	1,307 (31.3)	104 (29.3)	0.80 (0.59-1.08)	0.79 (0.58-1.07)
P-trend			0.21	0.18
**CVD specific-mortality**		**N=400**		
**BMI**				
Continuous (per 5 kg/m^2)	4,177 (100)	400 (100)	1.31 (1.18, 1.45)	1.31 (1.18, 1.45)
Categorical (kg/m^2)				
BMI<25	2,059 (48.0)	179 (44.8)	1.00 (Ref.)	1.00 (Ref.)
25≤BMI<30	1,364 (31.8)	124 (31.0)	1.09 (0.86-1.37)	1.07 (0.85-1.35)
BMI≥30	754 (18.1)	97 (24.2)	1.96 (1.51-2.53)	1.98 (1.53-2.56)
P-trend			<0.001	<0.001
**Walking (hours/week)**				
Categorical (hours/week)				
<1	808 (19.3)	84 (20.4)	1.00 (Ref.)	1.00 (Ref.)
1-≤3	2,019 (48.4)	202 (49.1)	0.91 (0.70-1.18)	0.92 (0.71-1.20)
≥4	1,307 (31.3)	105 (26.2)	0.81 (0.60-1.10)	0.82 (0.61-1.11)
P-trend			0.16	0.18

BC: Breast cancer. CVD: Cardiovascular disease. BMI: Body mass index. HR: Hazard ratio. CI: confidence interval.

© 2026 Bodelon C et al. *JAMA Network Open*.

[a]Models for BMI as the main exposure were adjusted for age at diagnosis (continuous), education (high school or less, some college/vocational school, college graduate, graduate school, unknown), smoking (never, former, current, unknown), and alcohol (not current drinker, <1 drink per week, 1-6 drinks per week, 1 drink per day, ≥2 drinks per day, unknown). Models with walking as the main exposures were adjusted for all the above variables and BMI (continuous).
[b]Models were additionally adjusted for estrogen receptor status, stage, receipt of chemotherapy, receipt of radiation therapy and receipt of endocrine therapy.

© 2026 Bodelon C et al. *JAMA Network Open*.

eTable 2. Associations of the BMI-PGS With BC-Specific and CVD-Specific Mortality

**BMI-PGS**	**Num. of BC survivors (N=4,177) N (%)**	**Num. of deaths N (%)**	**HR (95% CI)[a]**
		BC-specific deaths (N=355)	
Continuous (per 1-SD)	4,177 (100)	355 (100)	1.06 (0.96-1.18)
Tertiles			
1	1,392 (33.3)	108 (30.4)	1.00 (ref.)
2	1,392 (33.3)	130 (36.6)	1.26 (0.98-1.63)
3	1,393 (33.3)	118 (33.0)	1.15 (0.89-1.50)
		CVD-specific deaths (N=400)	
Continuous (per 1-SD)	4,177 (100)	400 (100)	1.06 (0.97-1.18)
Tertiles			
1	1,392 (33.3)	133 (33.2)	1.00 (ref.)
2	1,392 (33.3)	130 (32.5)	1.04 (0.81-1.32)
3	1,393 (33.3)	137 (34.2)	1.08 (0.85-1.38)

BMI: Body mass index. GRS: Genetic risk score. BC: Breast cancer. sd: Standard deviation. CVD: Cardiovascular disease. HR: Hazard ratio. CI: confidence interval.

[a]Adjusted for age (continuous), first five principal components for population stratification, and genotyping chip array.

© 2026 Bodelon C et al. *JAMA Network Open*.

eFigure 3. Causal Diagram of the Relationship Between the BMI-PGS, BMI, and All-Cause Mortality

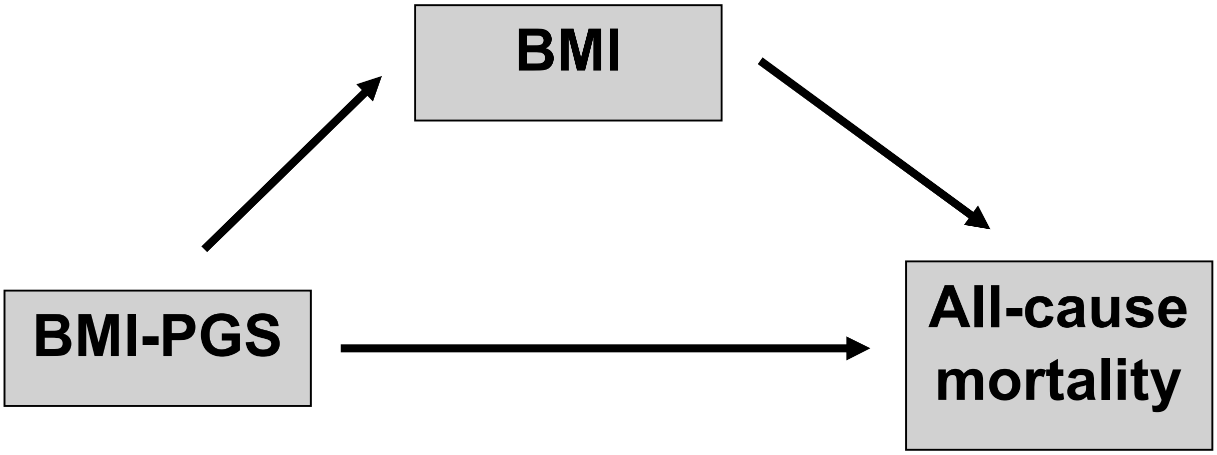

© 2026 Bodelon C et al. *JAMA Network Open*.
