# Supplementary material for: Genetic Predisposition to Excess Body Weight and Survival in Women Diagnosed With Breast Cancer
Source: JAMA Netw Open. 2026 Jan 13;9(1):e2553687. doi: 10.1001/jamanetworkopen.2025.53687 (PMC12801084; doi:10.1001/jamanetworkopen.2025.53687)
Supplement: Supplement 2. — Data Sharing Statement [file jamanetwopen-e2553687-s002.pdf]

Data Sharing Statement

Bodelon. Genetic Predisposition to Excess Body Weight and Survival in Women Diagnosed With Breast Cancer. *JAMA Netw Open*. Published January 13, 2026.
doi:10.1001/jamanetworkopen.2025.53687

Data
Data available: No

Additional Information
Explanation for why data not available: Data are available from the American Cancer Society (ACS) by following the ACS Data Access Procedures (https://www.cancer.org/content/dam/cancer-org/research/epidemiology/cancer-prevention-study-data-access-policies.pdf) for researchers who meet the criteria for access to confidential data. Please email cohort.data@cancer.org to inquire about access.
